# Supplementary material for: Content-rich biological network constructed by mining PubMed abstracts
Source: BMC Bioinformatics. 2004 Oct 8;5:147. doi: 10.1186/1471-2105-5-147 (PMC528731; doi:10.1186/1471-2105-5-147)
Supplement: Additional File 5 — The original Chilibot query results of the term "long-term potentiation (LTP)" and 22 other terms, limiting the latest references analyzed to the years 1990, 1995, 2000, and 2004. [file 1471-2105-5-147-S5.bz2 › chilibotAdditionalFile5/ltp2004/html/PKC_ACTININ.html]

 


 **PKC** and **ACTININ** 
  
Found 9 abstracts in PubMed,  **9 abstracts were retrieved and analyzed**.  


---

 Search Google  |
 PDF files only 
|  EDU domain only 

---

**Interactive relationship** (e.g. stimulation, inhibition, etc)

- Vinculin but not alpha  **actinin**  is a target of  **PKC**  phosphorylation during junctional assembly induced by calcium.  Ref: 9811570 J Cell Sci, 1998
- Treatment of cells with the  **PKC**  inhibitor calphostin C inhibited spreading and the colocalization of talin, alpha  **actinin** , tensin, and actin for both alpha v beta 3 and alpha v beta 5.  Ref: 8794871 J Cell Biol, 1996
- Chemotactic peptide, activators of  **PKC** , and cell adhesion all induce a significant increase in the amount of cytoskeletal alpha  **actinin**  and actin.  Ref: 10413608 Exp Cell Res, 1999

- :-)

  **Parallel relationship** (e.g. studied together, co-existance, homology, etc.)

  - **PKC**  epsilon GFP bound with a striated pattern that co localized with alpha  **actinin** , a marker of the Z line of the sarcomere.  Ref: 11325717 Biophys J, 2001
  - Activation of  **PKC**  by adding phorbol ester to alpha v beta 5 expressing cells induced spreading, increased colocalization of alpha  **actinin** , tensin, vinculin, p130cas and actin, and triggered tyrosine phosphorylation of FAK.  Ref: 8794871 J Cell Biol, 1996
  - Staining of  **PKC**  and vimentin, alpha  **actinin** , and vinculin suggests that  **PKC**  participates actively in the transduction of mechanical signals to the cell through focal adhesions and the cytoskeleton, although only  **PKC**  seemed to change with short time periods of strain.  Ref: 7942169 J Bone Miner Res, 1994
  - Immunolocalization of the cytoskeleton proteins vimentin and alpha  **actinin** , focal contact protein vinculin, and  **PKC**  showed a marked difference between strained and nonstrained cells.  Ref: 7942169 J Bone Miner Res, 1994
  - We report a selective, differential stimulus dependent enrichment of the actin associated protein alpha  **actinin**  and of isoforms of the signaling enzyme protein kinase C  **PKC**  in the neutrophil cytoskeleton.  Ref: 10413608 Exp Cell Res, 1999
